# Magnetic resonance imaging patterns of paediatric brain infections: a pictorial review based on the Western Australian experience

**DOI:** 10.1186/s13244-022-01298-1

**Published:** 2022-10-04

**Authors:** Chi-Wei Robin Yang, Michael Mason, Paul M. Parizel, Richard Warne

**Affiliations:** 1 Department of Medical Imaging, Perth Children’s Hospital (PCH), 15 Hospital Avenue, Nedlands, WA 6009 Australia; 2 Department of Radiology, University of Western Australia (UWA), Royal Perth Hospital (RPH), Perth, WA Australia

**Keywords:** Infectious encephalitis, Viral encephalitis, Differential diagnosis, Diagnostic imaging, Magnetic resonance imaging

## Abstract

Paediatric brain infections are relatively uncommon, but it is important for radiologists to recognise the disease and provide accurate differential diagnoses. Magnetic resonance imaging (MRI) plays an important role in determining the most likely causative pathogen—either in the setting of an unwell child with acute infectious encephalitis, or in the evaluation of a child for sequela of prior infection. Image analysis can, however, be difficult since a particular pathogen can cause variable MRI findings across different geographic environments, and conversely, a particular appearance on MRI may be caused by a variety of pathogens. This educational review aims to identify some of the key MRI patterns seen in paediatric brain infections and present example cases encountered in Western Australia. Based on (i) the predominant type of signal abnormality (restricted diffusion versus T2 hyperintensity) and (ii) the distribution of signal abnormality throughout the brain, this review presents a framework of six key MRI patterns seen in paediatric brain infections, with an emphasis on acute infectious encephalitis. There is general utility to these MRI patterns—each suggestive of a group of likely diagnostic possibilities which can be calibrated according to institution and local environment. The pattern-based framework of this review can be easily transitioned into daily radiological practice, and we hope it facilitates the formation of accurate differential diagnoses in paediatric brain infections.

**Supplementary Information:**

The online version contains supplementary material available at 10.1186/s13244-022-01298-1.

**Key points**

- There are several key MRI patterns in the setting of paediatric brain infections, which are common across geographic boundaries.
- Mechanism of dissemination (such as haematogenous or neural spread) and patient age (maturity of immune system) contribute to imaging appearances.
- Patterns based on abnormal restricted diffusion can manifest primarily in supratentorial white matter, supratentorial grey matter, or in the corpus callosum.
- Patterns based on abnormal high T2 signal can manifest primarily in supratentorial white matter, the basal ganglia/thalami, or the posterior fossa.
- Each pattern suggests a group of differential diagnoses, which can be calibrated according to institution and geographic environment.

## Background

Paediatric brain infections are an uncommon but important disease group. Through the use of magnetic resonance imaging (MRI), radiologists are central to the process of establishing differential diagnoses—either in the setting of an unwell child with acute infectious encephalitis (acute inflammation of brain parenchyma) or in the evaluation of a child for sequela of prior infection. However, establishing an accurate differential diagnosis can be challenging, as there are heterogeneous MRI findings described in the literature. A particular pathogen can cause variable brain MRI findings across different geographic environments, and a particular appearance on MRI may be caused by a variety of pathogens. For example:

- *Burkholderia pseudomallei*, a gram negative aerobic bacterium found in tropical and subtropical areas, manifests primarily as supratentorial brain abscesses in Southeast Asia due to bacteraemic spread following ingestion of contaminated food/water [1]. In comparison, *Burkholderia* manifests as rhomboencephalitis in Northern Australia, relating to nasopharyngeal mucosal colonisation during the wet season, followed by retrograde spread of bacteria to the brainstem [1].
- The pattern of restricted diffusion in deep and periventricular white matter (with a radiating pattern which appears to follow the deep medullary veins) is a well-documented finding in neonatal viral encephalitis, albeit across different geographic environments—such as by rotavirus in Korea and by Chikungunya virus in the Reunion Islands [2, 3].

Although these examples highlight the importance of reviewing regional datasets so that differential diagnoses can be tailored to the local environment, they also reveal the existence of imaging patterns which are common across geographic boundaries. The purpose of this educational review is to illustrate some of the key patterns of brain infections as seen on MRI, describe possible pathophysiologic mechanisms for these patterns, and present example cases encountered in Western Australia. The emphasis is on patterns of acute infectious encephalitis, although patterns relating to post-infectious sequela will also be discussed. Non-infectious aetiologies, such as autoimmune and metabolic conditions, have not been the focus of this review. Whilst other educational reviews on brain infections may present information categorised by microbial types, the structure of this review is to document a pattern-based framework that can be useful for narrowing the differential diagnosis and can be easily implemented into daily radiological practice.

## Key patterns of paediatric brain infections on MRI

The MRI patterns and example cases presented in this review are derived from a set of 95 microbiology-proven cases of brain infection in Western Australia, corroborated with the findings of published literature. Patient cases were identified through retrospective analysis of MRI and microbiology data for children treated at Perth Children’s Hospital and Prince Margaret Hospital for Children in Western Australia (WA) between the start of 2010 and March 2021. Ethics approval was obtained through the Western Australian Governance, Evidence, Knowledge and Outcomes (GEKO) system. The mean age of patients included in the data set was 2 years and 6 months (range 0 months through to 15 years and 9 months), with a similar gender distribution (51 males versus 44 females). Upon analysis of MRI cases, the following two factors were found to be of the highest diagnostic value in determining the causative pathogen in paediatric brain infection:

- The predominant type of signal abnormality (restricted diffusion versus T2 hyperintensity)
- The distribution pattern of signal abnormalities throughout the brain

Based on the above factors, six key MRI patterns relating to paediatric brain infection were identified, as listed below:

1. Restricted diffusion in supratentorial white matter
2. Restricted diffusion in supratentorial grey matter
3. Restricted diffusion in corpus callosum
4. T2 hyperintensity in supratentorial white matter
5. T2 hyperintensity in the basal ganglia and/or thalami
6. T2 hyperintensity in the posterior fossa

**Fig. 1:**
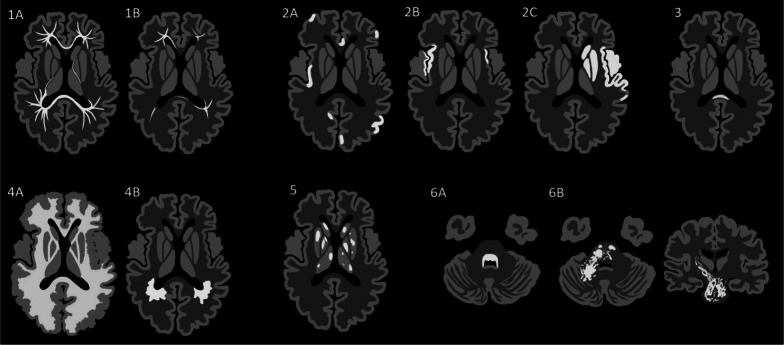
General MRI patterns of paediatric brain infections. Composite images of disease patterns overlaid on generic axial sections through the supratentorial brain (level of basal ganglia and third ventricle) and infratentorial brain (mid-pons and 4th ventricle), as well as a coronal section through the basal ganglia and third ventricle. Each key pattern on MRI has been allocated a number, with subdivisions indicated by a letter. Pattern 1: Restricted diffusion in supratentorial white matter (A—diffuse, B—limited). Pattern 2: Restricted diffusion in supratentorial grey matter injury (A—scattered, B—mesial temporal lobe(s), C—vascular territory). Pattern 3: Restricted diffusion in corpus callosum. Pattern 4: T2 hyperintensity in supratentorial white matter (A—with neuronal migration abnormality, B—without neuronal migration abnormality). Pattern 5: T2 hyperintensity in the basal ganglia and/or thalami. Pattern 6: T2 hyperintensity in the posterior fossa (A—dorsal pons, B—diffuse brainstem with longitudinal tract involvement)

Using generic axial and coronal templates of the brain, stylised images representing each pattern were hand drawn using graphics software (following a format inspired by de Oliveira et al.’s incisive article on toxic and metabolic brain disorders) [4]. These stylised images, along with descriptions of sub-patterns, are detailed in Fig. 1. The pathogens encountered in Western Australia, pertaining to each pattern and sub-pattern, are summarised in Table 1—discrepancy between the number of patterned cases and number of microbiology-proven cases of brain infection relates to the large proportion of microbiology-proven cases (particularly of viral aetiology) with normal or near-normal MRI studies.

**Table 1:** Summary of pathogens encountered in Western Australia, pertaining to each pattern

Pattern	Cases	Pathogens
**1**		
A	6	Parechovirus (5 cases), Enterovirus (1 case)
B	6	Parechovirus (2 cases), Enterovirus (4 cases)
**2**		
A	7	Herpes simplex virus (5 cases), Scedosporium prolificans (1 case), Pneumocystis jiroveci (1 case)
B	1	Herpes simplex virus (1 case)
C	2	Varicella zoster virus (1 case), Mycobacterium tuberculosis (1 case)
**3**		
	1	Norovirus (1 case)
**4**		
A	7	Cytomegalovirus (5 cases), Toxoplasma (2 cases)
B	6	Cytomegalovirus (6 cases)
**5**		
	4	Epstein-Barr virus (2 cases), Cryptococcus (2 cases)
**6**		
A	3	Enterovirus (3 cases)
B	1	Burkholderia pseudomallei (1 case)

In the following sections, each pattern is discussed in further detail, with exploration of possible pathophysiologic mechanisms, the types of causative pathogens (with example cases), and corroboration with the published literature.

### Pattern 1: Restricted diffusion in supratentorial white matter

**Fig. 2:**
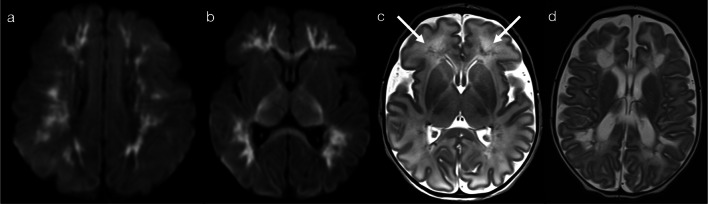
Restricted diffusion in supratentorial white matter—diffuse (Pattern 1A). 2-day-old term infant with generalised seizures and CSF-proven parechovirus encephalitis. DWI (**a**, **b**) demonstrated a striking pattern of restricted diffusion involving deep and periventricular white matter. On T2-weighted imaging (**c**), corresponding foci of low signal intensity were relatively subtle (arrows). A follow-up scan 5 weeks later (**d**) revealed significant tissue loss with areas of cystic encephalomalacia

**Fig. 3:**
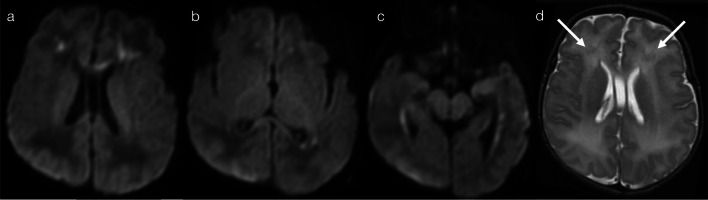
Restricted diffusion in supratentorial white matter—limited (Pattern 1B). 8-day-old term infant with raised inflammatory markers and vomiting (no seizures). CSF was positive for enterovirus. DWI (**a**–**c**) demonstrated scattered foci of restricted diffusion involving deep and periventricular white matter, best seen in the frontal lobes (**a**) and temporal lobes (**c**). On T2-weighted imaging (**d**), corresponding foci of low signal intensity were difficult to appreciate (arrows)

The presence of restricted diffusion typically indicates cytotoxic oedema, with less common causes including high viscosity and high cellularity (as seen with pyogenic abscesses) [5]. The mechanism for restricted diffusion which bilaterally and relatively symmetrically involves deep and periventricular white matter, with a radiating pattern which appears to follow the deep medullary veins, that is not fully understood. Potential mechanisms include neuroaxonal tropism (activation of toll-like receptors and subsequent inflammatory response), perivenular invasion or venous ischaemia [6]. Nevertheless, in neonates, two important pathogens implicated in this pattern are parechovirus and enterovirus infection [6–8]. The cytotoxic oedema seen on diffusion-weighted imaging (DWI) is the predominant imaging feature, with corresponding signal abnormalities on T1 (hyperintensity)- and T2 (hypointensity)-weighted imaging being relatively subtle [6]. The extent of restricted diffusion can range from florid (pattern 1A—Fig. 2) to mild (pattern 1B—Fig. 3), and cystic encephalomalacia can be seen as a sequelae of severe white matter injury [7]. Rotavirus, adenovirus, Chikungunya and herpes simplex virus (HSV) have also been described in the literature as producing similar appearances on DWI in neonates [2, 3, 9, 10]. As a memory aid, the differential list of parechovirus, adenovirus, rotavirus, enterovirus, Chikungunya and HSV conveniently spells the mnemonic P-A-R-E-C-H.

### Pattern 2: Restricted diffusion in supratentorial grey matter

Cytotoxic oedema involving grey matter, as seen on DWI, represents a heterogeneous group which can be divided into three sub-patterns for the purposes of this review.

**Fig. 4:**
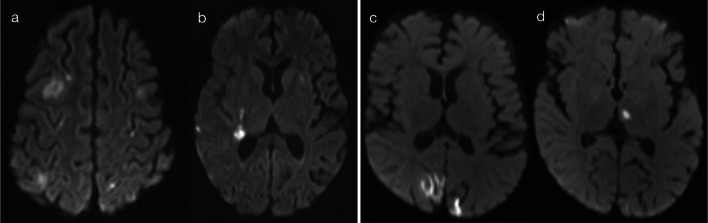
Restricted diffusion in supratentorial grey matter—scattered lesions (Pattern 2A). Case 1: 4-year-old child with somnolence and hyperaesthesia, in the setting of acute lymphoblastic leukaemia (ALL) and *Scedosporium prolificans* fungaemia. DWI (**a**, **b**) demonstrated scattered foci of restricted diffusion, mainly affecting cortical grey matter with a bilateral though asymmetric distribution. Case 2: 4-month-old infant with respiratory distress, in the setting of HIV, *Pneumocystis jiroveci* pneumonia and an abnormal cranial ultrasound. DWI (**c**, **d**) demonstrated scattered foci of restricted diffusion, mainly affecting cortical grey matter with a bilateral though asymmetric distribution

**Fig. 5:**
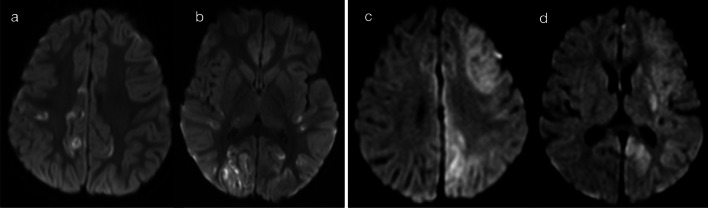
Restricted diffusion in supratentorial grey matter—scattered lesions (Pattern 2A). Case 1: 3-year-old child with fever, headaches and reported visual loss. CSF was positive for HSV-1. DWI (**a**, **b**) demonstrated foci of restricted diffusion in cortical grey matter, with a bilateral though asymmetric distribution. Case 2: 10-month-old infant with afebrile focal seizures. CSF was positive for HSV-1. DWI (**c**, **d**) demonstrated regions of confluent cortical restricted diffusion, with an asymmetric distribution

In the first sub-pattern, mainly observed in infants and young children, scattered and asymmetric foci of restricted diffusion (pattern 2A) suggest haematogenous spread of disease. This could relate to occlusion/inflammation of small distal vessels as seen with septic emboli, particularly in immunocompromised children (Fig. 4) [11, 12], or alternatively with spread of viral particles across the immature blood brain barrier as seen with herpes simplex virus (HSV) infection (Fig. 5) [10, 13]. Early detection of HSV encephalitis and assessment of disease extent is best assessed on DWI [13, 14], although differentiation between early HSV encephalitis and septic emboli can be difficult. Progression of lesions (within days) to form confluent areas of cortical/subcortical signal abnormality is suggestive of HSV encephalitis [13–15], whereas abscess formation is consistent with septic emboli [11, 12].

**Fig. 6:**
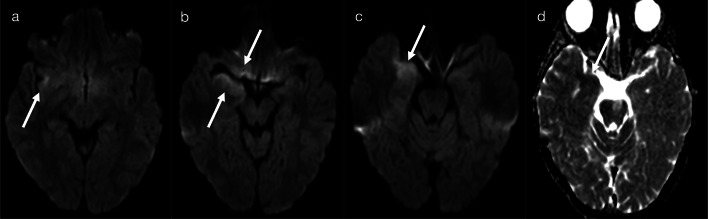
Restricted diffusion in supratentorial grey matter—mesial temporal lobe (Pattern 2B). 7-year-old child with headache, fluctuating consciousness and tonic–clonic seizures. CSF was positive for HSV-1. DWI (**a**–**c**) and ADC map (**d**) demonstrated asymmetric signal abnormalities predominantly involving the right mesial temporal lobe (amygdala and uncus), anterior perforated substance and insular cortex (arrows), as would be typical for HSV encephalitis in adults

A second sub-pattern of supratentorial grey matter diffusion restriction in infants and young children corresponds to neural spread of disease, as opposed to the previously described haematogenous spread. As can be observed with HSV in older children, cytotoxic oedema can affect the mesial temporal and insular cortices (pattern 2B—Fig. 6), relating to spread of viral particles along meningeal branches of the trigeminal ganglion [10, 14].

**Fig. 7:**
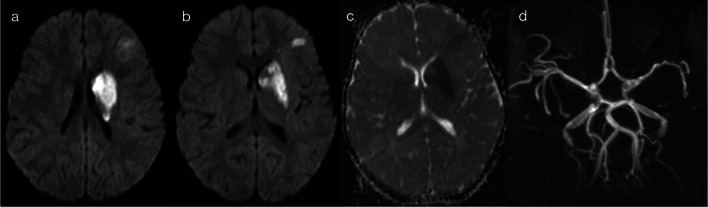
Restricted diffusion in supratentorial grey matter—vascular territory infarct (Pattern 2C). 2-year-old child with right upper and lower limb weakness in the setting of prior varicella infection (IgG positive), in keeping with post-varicella arteriopathy. DWI (**a**, **b**) and apparent diffusion coefficient (ADC) map (**c**) were consistent with an acute left caudato-lenticular infarct and a small infarct within the left frontal lobe. MR angiography (**d**) demonstrated left middle cerebral arteriopathy

**Fig. 8:**
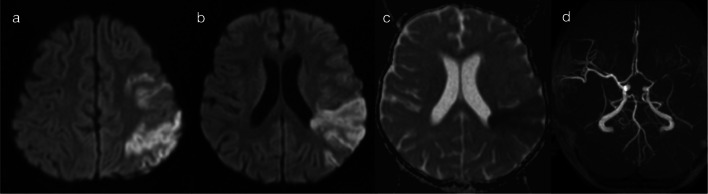
Restricted diffusion in supratentorial grey matter—vascular territory infarct (Pattern 2C). 4-year-old child with 2 weeks of malaise, followed by decreased conscious state and right arm weakness. CSF was positive for tuberculosis (TB). DWI (**a**, **b**) and ADC map (**c**) were consistent with an acute infarct involving the left frontoparietal lobes. MR angiography (**d**) demonstrated left middle cerebral arteriopathy

In a third sub-pattern of supratentorial grey matter diffusion restriction, infection can lead to ischaemic stroke, resulting in cytotoxic oedema (restriction on DWI) in distinct vascular territories (pattern 2C). Although not an infectious encephalitis per se, the child will nevertheless present unwell with acute neurological signs, and it is important to recognise the role of recent infection in the child’s presentation. The chicken pox virus is a notable cause, leading to post-varicella arteriopathy (Fig. 7) [16, 17]. Microbes such as HSV, EBV, enterovirus and TB (Fig. 8) have also been implicated in the literature [16, 17].

### Pattern 3: Restricted diffusion in corpus callosum

**Fig. 9:**
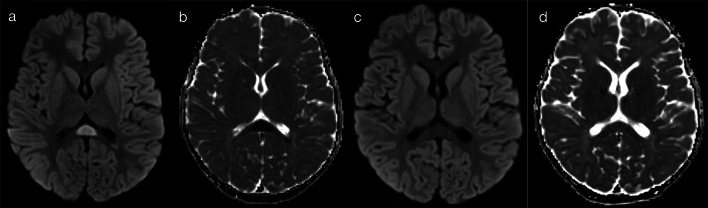
Restricted diffusion in corpus callosum (Pattern 3). 3-year-old child with gastrointestinal illness, followed by lethargy, irritability and suspected focal seizures. Stool was positive for norovirus. DWI (**a**) and ADC map (**b**) demonstrated a cytotoxic lesion midline within the splenium. Follow-up MRI 2 weeks later demonstrated complete resolution of the splenial lesion on DWI (**c**) and ADC (**d**) images

Cytotoxic lesions of the corpus callosum (CLOCCs) are encountered in a number of settings, including infection, inflammation and trauma; in children, the most common cause is infection [18, 19]. The vulnerability of the corpus callosum, in particular the splenium, is thought to relate to an increased number of cytokine (and ultimately glutamate) receptors, which in conjunction with its rich blood supply (from both anterior and posterior circulations) makes the corpus callosum vulnerable to cytokinopathy in settings such as infection [19, 20]. Whilst various patterns of callosal involvement have been described, the most common pattern in children is an ovoid lesion centred midline within the splenium (Fig. 9) [18, 19]. Typical infectious agents implicated in CLOCCS include influenza (most common), herpesviridae and gastrointestinal pathogens (e.g. rotavirus, *Escherichia coli* and *Salmonella enteritis*) [18–20]. In the paediatric cohort, the reversibility of CLOCCS (within 1–2 weeks) typically confers a favourable prognosis; conversely, persistence of restricted diffusion within the corpus callosum should prompt consideration of non-infectious aetiologies (e.g. metabolic or traumatic) [18].

### Pattern 4: T2 hyperintensity in supratentorial white matter

Diffuse or confluent supratentorial white matter T2 hyperintensities, in the absence of DWI changes related to cytotoxic oedema, suggest abnormalities such as gliosis and/or encephalomalacia (as the end-product of prior or longstanding infection) [21, 22]. In the neonatal setting, congenital TORCH infections (toxoplasma, other, rubella, cytomegalovirus and herpes simplex virus) come to mind as important differentials with manifestations in supratentorial white matter [21–23]. The presence of calcification is likewise an indicator of brain parenchymal injury during the early stages of life, arising from parenchymal necrosis in conjunction with an immature immune system and the impaired phagocytic ability of macrophages [23].

**Fig. 10:**
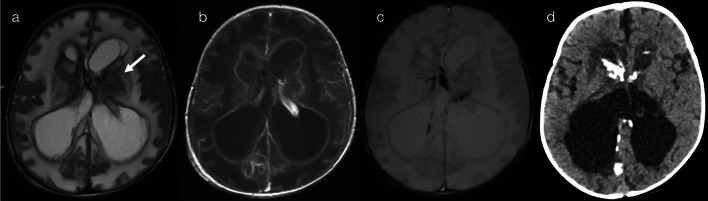
T2 hyperintensity in supratentorial white matter—with neuronal migration abnormality (Pattern 4A). 4-week-old infant with seizures, irritability and bulging fontanelles. CSF analysis was consistent with neurotoxoplasmosis. T2-weighted imaging (**a**) demonstrated diffuse white matter hyperintensity, widespread abnormal cortical development and hydrocephalus. Lesions were seen throughout the cerebral hemispheres, in the periventricular regions (arrow), basal ganglia and the thalami. Post-contrast T1-weighted imaging (**b**) demonstrated peripheral enhancement of parenchymal lesions, and ependymal enhancement in the lateral ventricles (reflecting ventriculitis). On a T2*-weighted sequence (**c**), foci of susceptibility artefact corresponded to areas of haemorrhage and calcification (secondary to parenchymal necrosis). Non-contrast CT head performed 5 months later (**d**) better demonstrated regions of periventricular calcification

**Fig. 11:**
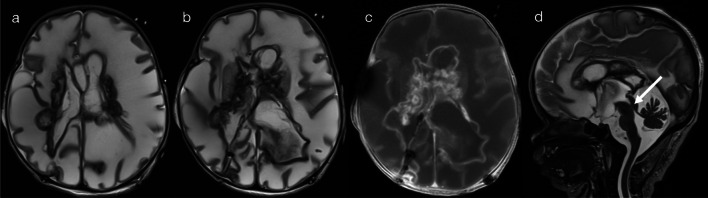
T2 hyperintensity in supratentorial white matter—with neuronal migration abnormality (Pattern 4A). 2-week-old infant with multiple seizures and a bulging anterior fontanelle. CSF analysis was consistent with neurotoxoplasmosis. T2-weighted images (**a**, **b**, **d**) demonstrated diffuse white matter injury, widespread abnormal cortical development and hydrocephalus relating to aqueduct stenosis (arrow). Post-contrast T1-weighted imaging (**c**) demonstrated multiple peripherally enhancing lesions in the basal ganglia and periventricular regions, as well as enhancement of the ependymal lining of the lateral ventricles (reflecting ventriculitis)

**Fig. 12:**
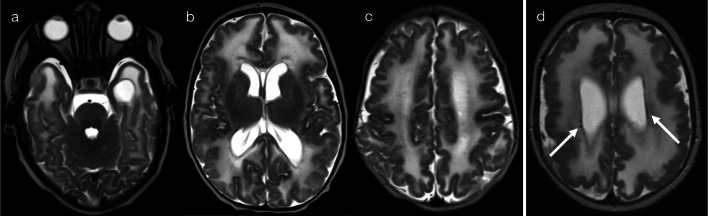
T2 hyperintensity in supratentorial white matter—with neuronal migration abnormality (Pattern 4A). Case 1: 11-week-old infant with bilateral sensorineural hearing loss on newborn testing. Guthrie test was positive for CMV. T2-weighted imaging (**a**–**c**) demonstrated polymicrogyria with diffuse white matter hyperintensity and cystic change in the left anterior temporal pole. Clinical notes at 14 months of age indicated developmental delay. Case 2: 4-month-old infant with microcephaly, developmental delay and hypertonia under investigation. Guthrie test was positive for CMV. T2-weighted imaging (**d**) demonstrated polymicrogyria with diffuse white matter hyperintensity and subtle periventricular calcifications (arrows)

**Fig. 13:**
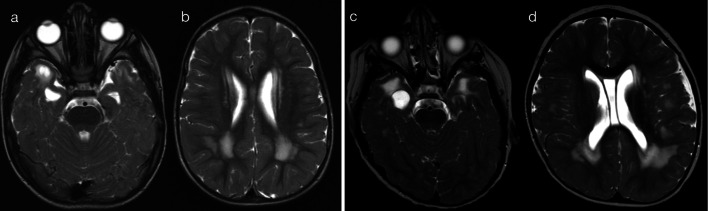
T2 hyperintensity in supratentorial white matter—without neuronal migration abnormality (Pattern 4B). Case 1: 2-year-old child with congenital left sensorineural hearing loss. Guthrie test was positive for CMV. T2-weighted imaging (**a**, **b**) demonstrated cystic change at the right anterior temporal pole, with bilateral parietal and periventricular white matter hyperintensity. Case 2: 2-year-old child with developmental delay. Guthrie test was positive for CMV. T2-weighted imaging (**c**, **d**) demonstrated cystic change at the right anterior temporal pole, with bilateral peritrigonal white matter hyperintensity

The timing of TORCH infection is central to its imaging manifestations [23]. Early in-utero infection, such as during the early stages of the second trimester of pregnancy, is more likely to result in malformations of cortical development (lissencephaly-pachygyria or polymicrogyria), brain volume loss and diffuse white matter abnormality (pattern 4A—Figs. 10, 11 and 12) [23–26]. Conversely, TORCH infection during late third trimester of pregnancy is seen without neuronal migration abnormality, and any long-term neurodevelopmental sequelae are typically less severe (pattern 4B—Fig. 13) [23–25]. Although post-natal imaging depicts the sequela of infection rather than ongoing infectious encephalitis, being able to differentiate between early and late in utero TORCH infection has important prognostic implications, and establishes the utility of MRI in the work-up of infants and young children with abnormal neurology/development.

Whilst there is overlap in the imaging features of different TORCH infections, certain findings can help distinguish between cases of congenital neurotoxoplasmosis and cytomegalovirus (CMV) infection. Congenital neurotoxoplasmosis (Figs. 10 and 11) is characterised by hydrocephalus, parenchymal volume loss, necrosis with abscess formation, and calcifications (typically coarse and random in distribution); chorioretinitis with vision impairment is a supportive clinical feature [23, 26]. In comparison, congenital CMV (Figs. 12 and 13) is characterised by anterior temporal pole cysts, parietal/peritrigonal white matter T2 hyperintensity, microcephaly and calcifications (typically periventricular); sensorineural hearing loss is a supportive clinical feature, and may be the initial trigger for investigation [24–26].

When there is supratentorial white matter hyperintensity and the typical peritrigonal distribution of CMV is not observed (in an otherwise developmentally normal brain), non-TORCH infections should be considered. For example, *Streptococcus pneumoniae* can cause diffuse white matter injury involving the centrum semiovale bilaterally [27]. Non-infectious differentials for the anterior temporal pole cysts seen in congenital CMV include megalencephalic leukoencephalopathy with subcortical cysts (MLC) and Aicardi-Goutières syndrome (AGS) [25].

### Pattern 5: T2 hyperintensity in the basal ganglia and/or thalami

A number of pathogens exhibit tropism for the basal ganglia and/or thalami, including Epstein-Barr virus (EBV), varicella zoster virus (VZV), Flaviviridae (such as Dengue virus, West Nile virus, Murray Valley encephalitis virus and Japanese encephalitis virus), cryptococcus and tuberculosis [13, 23, 24, 28–32]. The mechanisms by which these pathogens affect deep brain structures is not fully understood, and may relate to a number of factors including the high inherent metabolic activity of the basal ganglia and thalami, in conjunction with their vascular supply [33]. In particular settings, such as with cryptococcal infection, infiltration of deep brain structures via perivascular spaces has been described [32, 34]. Post-inflammatory, genetic and metabolic causes should also be considered in pathology of the basal ganglia and thalami, and clinical history is fundamental [13, 28, 30, 31].

**Fig. 14:**
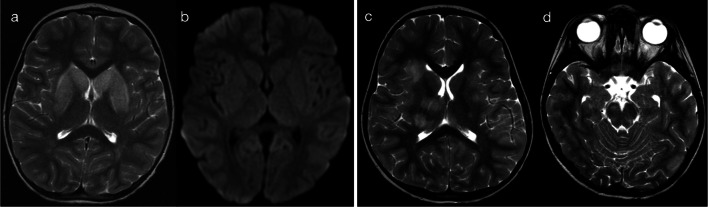
T2 hyperintensity in basal ganglia and/or thalami (Pattern 5). Case 1: 5-year-old child with afebrile generalised tonic–clonic seizures and CSF-proven EBV encephalitis. T2-weighted imaging (**a**) demonstrated bilateral hyperintensity of the basal ganglia, without abnormality on DWI (**b**). Case 2: 4-year-old child with fever, sore throat, irritability and CSF-proven EBV encephalitis. T2-weighted imaging (**c**, **d**) demonstrated bilateral hyperintensity of the basal ganglia and thalami (**c**), as well as scattered lesions in the cerebrum and brainstem (**d**). No DWI abnormality was present (not shown)

**Fig. 15:**
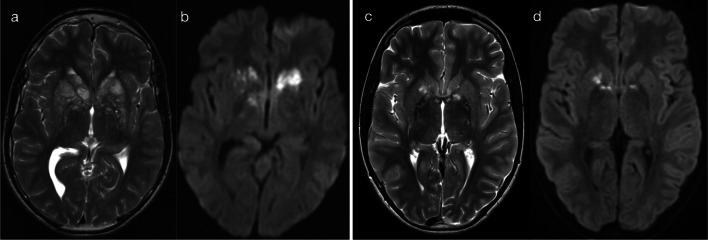
T2 hyperintensity in basal ganglia and/or thalami (Pattern 5). Case 1: 15-year-old child with headache, nausea and vomiting. CSF was positive for cryptococcus. T2-weighted imaging (**a**) demonstrated bubble-like lesions within the basal ganglia (relating to gelatinous pseudocysts), with a relatively smaller component of restricted diffusion (**b**). Case 2: 14-year-old child with malaise for 2 weeks. CSF was positive for cryptococcus. T2-weighted imaging (**c**) demonstrated bubble-like lesions within the basal ganglia and thalami, with a relatively smaller component of restricted diffusion (**d**)

EBV infections present with bilateral T2 hyperintense lesions of the basal ganglia and thalami, with variable symmetry, variable restricted diffusion and typically no gadolinium enhancement (Fig. 14) [13, 14, 23, 29]. Extension into the infratentorial brain may occasionally occur, and variable cortical grey matter involvement has been reported [23, 29]. Imaging features of cryptococcal infection reflect spread of disease along perivascular spaces, with formation of gelatinous pseudocysts predominantly in the basal ganglia—this gives rise to ‘bubble-like' lesions which are T2 hyperintense and may have a small component of restricted diffusion (Fig. 15) [24, 32]. Cases of flavivirus encephalitis were not available in our data set, but literature describes the presence of bilateral T2 hyperintensities in the thalami, with or without basal ganglia involvement [23, 30]. Although not known to be neurotropic for deep brain structures, respiratory pathogens (such as influenza virus, parainfluenza virus, respiratory syncytial virus, adenovirus and *Streptococcus pneumoniae*) have also been associated with lesions in the thalami (often symmetric), with a minority of cases leading to acute necrotising encephalitis (ANEC) [30, 35].

### Pattern 6: T2 hyperintensity in the posterior fossa

**Fig. 16:**
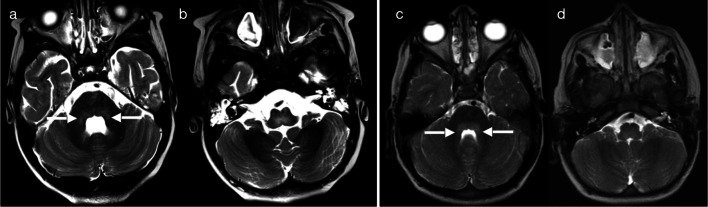
T2 hyperintensity in posterior fossa—dorsal pons (Pattern 6A). Case 1: 4-year-old child with fluctuating GCS. Stool was positive for enterovirus (serotype E-71). T2-weighted imaging (**a**, **b**) demonstrated hyperintensity of the dorsal pons and medulla, with extension of signal abnormality into the middle cerebellar peduncles bilaterally (arrows). Case 2: 3-year-old child with lethargy, vomiting and fever, as well as right facial nerve palsy and unsteadiness on feet. Stool was positive for enterovirus (serotype not known). T2-weighted imaging (**c**, **d**) demonstrated hyperintensity of the dorsal pons and medulla, with extension of signal abnormality into the middle cerebellar peduncles bilaterally (arrows)

**Fig. 17:**
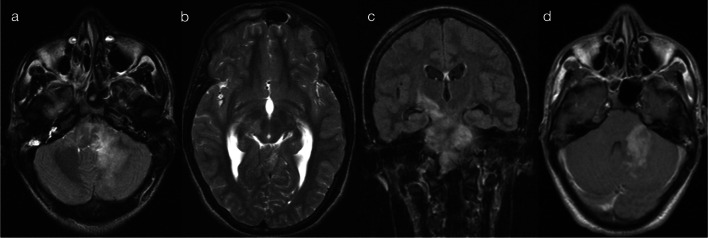
T2 hyperintensity in posterior fossa—diffuse brainstem with longitudinal tract involvement (Pattern 6B). 14-year-old child with headache, vomiting, left cerebellar signs, left upper motor neuron facial palsy, diplopia and slurred speech. T2-weighted images (**a**, **b**) demonstrated asymmetric T2 hyperintensity within the brainstem and left cerebellar hemisphere. Coronal FLAIR image (**c**) demonstrated extension of abnormality along white matter tracts to the contralateral supratentorial brain. Patchy enhancement was appreciable on post-contrast T1-weighted imaging (**d**). Minimal DWI change was seen (not shown). Neuromelioidosis was proved on brain tissue sampling

**Fig. 18:**
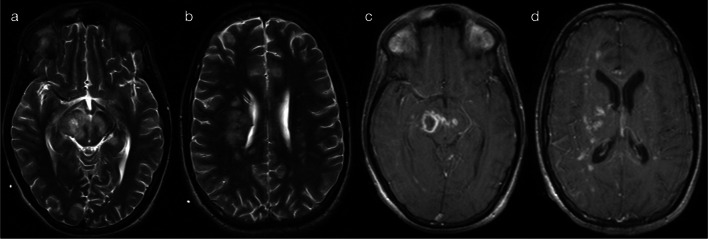
T2 hyperintensity in posterior fossa—diffuse brainstem with longitudinal tract involvement (Pattern 6B). Follow-up MRI after 2 weeks (same patient as Fig. 17 with biopsy-proven neuromelioidosis) showed T2 hyperintensities (**a**, **b**) following longitudinal white matter tracts into the supratentorial brain. Post-contrast T1-weighted imaging (**c**, **d**) demonstrated a rim-enhancing abscess in the right cerebral peduncle and multiple enhancing foci in the supratentorial brain (representing micro-abscesses)

Rhombencephalitis, with high T2 signal in the brainstem and/or cerebellum, can be associated with different infectious aetiologies—each with their own mechanism for affecting the posterior fossa. Enterovirus, which can initially present as a gastrointestinal illness or as a rash involving the hands and feet of infants and young children, spreads along peripheral nerves to gain access to the central nervous system where it has a predilection for ventral horn cells of the spinal cord and the brainstem [13]. When enterovirus causes rhombencephalitis, MRI typically demonstrates T2 hyperintensity of the dorsal pons and medulla oblongata (pattern 6A—Fig. 16), and there may be involvement of the midbrain, dentate nuclei and upper cervical cord (serotypes E-71 and E-68 are often cited as the causative agent) [36–39]. More specific to the Northern Australian setting, neuromelioidosis results from nasopharyngeal mucosal colonisation by *Burkholderia*, followed by retrograde spread to the brainstem to cause rhombencephalitis (cranial nerve deficits may be clinically apparent) and spread of micro-abscesses along longitudinal white matter and commissural tracts (pattern 6B—Fig. 17) [1, 40, 41]. Rim-enhancing brain abscesses may eventually develop in the posterior fossa and/or contralateral supratentorial compartment (Fig. 18) [1, 40, 41]. Although not encountered in our dataset, other pathogens implicated in rhombencephalitis include rotavirus, *Listeria monocytogenes* (e.g. from pre-cooked meats and unpasteurised milk), VZV and HSV [1, 42–45]. Viruses which affect the basal ganglia and thalami, such as EBV and flaviviridae, may also occasionally manifest in the posterior fossa [23, 28, 29].

## Conclusion

**Fig. 19:**
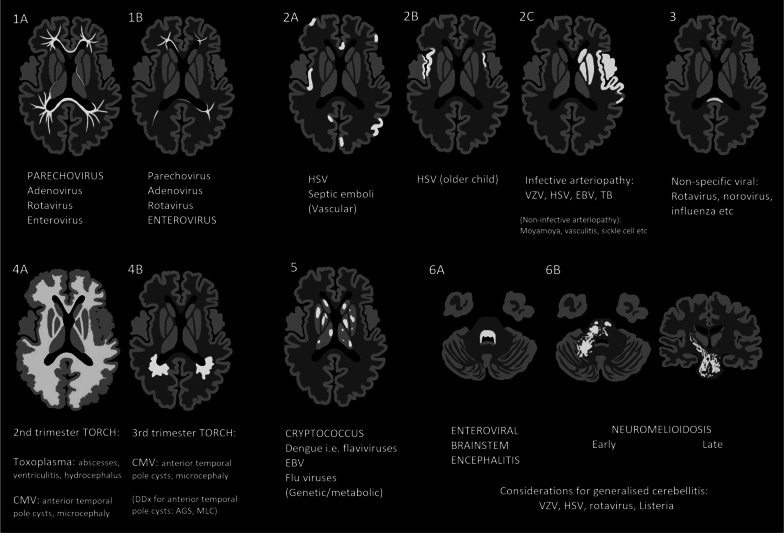
MRI patterns of paediatric brain infections, with differentials relevant to the WA environment. A summary of the key MRI patterns and their sub-patterns as discussed in this review, with the causative pathogens tailored to the WA environment

This educational review offers a practical framework for approaching paediatric brain infections. The key MRI patterns described in this review are each suggestive of a group of diagnostic possibilities—grounded in pathophysiology and corroborated by published literature, with flexibility for calibration according to institution and local environment (an example summary for Western Australia is shown as Fig. 19). The pattern-based framework of this review can be easily transitioned into daily radiological practice, and we hope it can facilitate the formation of accurate differential diagnoses in paediatric brain infections.

## Supplementary Information

**Additional file 1**. Supplementary figures with ADC maps.

## Data Availability

The dataset used and/or analysed during the current study are available from the corresponding author on reasonable request.

## Abbreviations

ADC: Apparent diffusion coefficient
AGS: Aicardi-Goutières syndrome
ANEC: Acute necrotising encephalitis
CLOCC: Cytotoxic lesion of the corpus callosum
CMV: Cytomegalovirus
DWI: Diffusion-weighted imaging
EBV: Epstein-Barr virus
HSV: Herpes simplex virus
MERS: Mild encephalitis with reversible splenial lesion
MLC: Megalencephalic leukoencephalopathy with subcortical cysts
MRI: Magnetic resonance imaging
TB: Tuberculosis
VZV: Varicella zoster virus
WA: Western Australia

## References

[1] McLeod C, Morris PS, Bauert PA (2015). Clinical presentation and medical management of melioidosis in children: a 24-year prospective study in the Northern Territory of Australia and review of the literature. CID.

[2] Yeom JS, Kim YS, Set JH (2015). Distinctive pattern of white matter injury in neonates with rotavirus infection. Neurology.

[3] Correa DG, Freddi TAL, Werner H (2020). Brain MR imaging of patients with perinatal chikungunya virus infection. AJNR Am J Neuroradiol.

[4] de Oliveira AM, Paulino MV, Vieira APF (2019). Imaging patterns of toxic and metabolic brain disorders. Radiographics.

[5] Finelli PF (2012). Diagnostic approach to restricted-diffusion patterns on MR imaging. Neurol Clin Prac.

[6] Sarma A, Hanzlik E, Krishnasarma R (2019). Human Parechovirus meningoencephalitis: neuroimaging in the era of polymerase chain reaction-based testing. AJNR Am J Neuroradiol.

[7] Verboom-Maciolek MA, Groenendaal F, Hahn CD (2008). Human parechovirus causes encephalitis with white matter injury in neonates. Ann Neurol.

[8] Verboon-Maciolek MA, Groenendaal F, Cowan F (2006). White matter damage in neonatal enterovirus meningoencephalitis. Neurology.

[9] Tamiya M, Komatsu H, Hirabayashi M (2019). Neonatal meningoencephalitis caused by human adenovirus species F infection. Pediatr Int.

[10] Baskin HJ, Hedlund G (2007). Neuroimaging of herpesvirus infections in children. Pediatr Radiol.

[11] Swinburne NC, Bansal AG, Agarwal A, Yoshi AH (2017). Neuroimaging in Central Nervous system infections. Curr Neurol Neurosci Rep.

[12] Rozell JM, Mtui E, Pan YN (2017). Infectious and inflammatory diseases of the central nervous system: the spectrum of imaging findings and differential diagnosis. Emerg Radiol.

[13] Moltoni G, D’arco F, Pasquini L (2020). Non-congenital viral infections of the central nervous system: from the immunocompetent to the immunocompromised child. Pediatr Radiol.

[14] Soares BP, Provenzale JM (2016). Imaging of herpesvirus infections of the CNS. AJR Am J Roentgenol.

[15] Leonard JR, Moran CJ, Cross DT (2000). MR imaging of herpes simplex type I encephalitis in infants and young children: a separate pattern of findings. AJR Am J Roentgenol.

[16] Mackay MT, Wiznitzer M, Benedict SL (2011). Arterial ischemic stroke risk factors: the international pediatric stroke study. Ann Neurol.

[17] Fullerton HJ, Elkins MSV, Barkovich AJ (2011). The vascular effects of infection in pediatric stroke (VIPS) study. J Child Neurol.

[18] Kontzialis M, Soares B, Huisman TA (2017). Lesions in the splenium of the corpus callosum on MRI in children: a review. J Neuroimaging.

[19] Starkey J, Kobayashi N, Numaguchi Y (2017). Cytotoxic lesions of the corpus callosum that show restricted diffusion: mechanisms, causes and manifestations. Radiographics.

[20] Tetsuka S (2019). Reversible lesion in the splenium of the corpus callosum. Brain Behav.

[21] Barnes PD (2001). Neuroimaging and the timing of fetal and neonatal brain injury. J Perinatol.

[22] Cheeran MC, Lokensgard JR, Schleiss MR (2009). Neuropathogenesis of congenital cytomegalovirus infection: disease mechanisms and prospects for intervention. Clin Microbiol Rev.

[23] Nickerson JP, Richner B, Santy K (2012). Neuroimaging of pediatric intracranial infection—Part 2: TORCH, viral, fungal, and parasitic infections. J Neuroimaging.

[24] Bhatia A, Pruthi S (2016). Imaging of pediatric infection within the central nervous system. Curr Radiol Rep.

[25] Fink KR, Thapa MM, Ishak GE (2010). Neuroimaging of pediatric central nervous system cytomegalovirus infection. Radiographics.

[26] Neuberger I, Garcia J, Meyers ML (2018). Imaging of neonatal central nervous system infections. Pediatr Radiol.

[27] Jorens PG, Parizel PM, Wojciechowski M (2008). Streptococcus pneumoniae meningoencephalitis with unusual and widespread white matter lesions. Eur J Paediatr Neurol.

[28] Maller VV, Bathla G, Moritani T (2017). Imaging in viral infections of the central nervous system: can images speak for an acutely ill brain?. Emerg Radiol.

[29] Abul-Kasim K, Palm L, Many P (2009). The neuroanatomic localization of epstein-barr virus encephalitis may be a predictive factor for its clinical outcome: a case report and review of 100 cases in 28 reports. J Child Neurol.

[30] Beattie GC, Glaser CA, Sheriff H (2013). Encephalitis with thalamic and basal ganglia abnormalities: etiologies, neuroimaging, and potential role of respiratory viruses. CID.

[31] Khanna PC, Iyer RS, Chaturvedi A (2011). Imaging bithalamic pathology in the pediatric brain: demystifying a diagnostic conundrum. AJR Am J Roentgenol.

[32] Xia S, Li X, Li H (2016). Imaging characterisation of cryptococcal meningoencephalitis. Radiol Inf Dis.

[33] Hedge AN, Mohan S, Lath N (2011). Differential diagnosis for bilateral abnormalities of the Basal Ganglia and Thalamus. Radiographics.

[34] Shih RY, Koeller KK (2015). Bacterial, Fungal, and parasitic infections of the central nervous system: radiologic-pathologic correlation and historical perspectives. Radiographics.

[35] Magnus J, Parizel PM, Ceulemans B (2011). Streptococcus pneumoniae meningoencephalitis with bilateral basal ganglia necrosis: an unusual complication due to vasculitis. J Child Neurol.

[36] Shen WC, Chiu HH, Chow KC (1999). MR imaging findings of enteroviral encephalomyelitis: an outbreak in Taiwan. AJNR Am J Neuroradiol.

[37] Abdelgawad MS, El-Nekidy AE, Abouyoussef RA (2016). MRI findings of enteroviral encephalomyelitis. Egypt J Radiol Nucl Med.

[38] Fan YK, Liu YP (2019). Magnetic resonance imaging features of pediatric coxsackievirus encephalitis. J Belg Soc Radiol.

[39] Maloney JA, Mirsky DM, Messacar K (2014). MRI findings in children with acute flaccid paralysis and cranial nerve dysfunction occurring during the 2014 enterovirus D68 outbreak. AJNR Am J Neuroradiol.

[40] Hsu CC, Singh D, Kwan G (2016). Neuromelioidosis: craniospinal MRI findings in Burkholderia pseudomallei Infection. J Neuroimaging.

[41] Wongwandee M, Linasmita P (2019). Central nervous system melioidosis: A systematic review of individual participant data of case reports and case series. PLoS Negl Trop Dis.

[42] Bozzola E, Bozzola M, Tozzi AE (2014). Acute cerebellitis in varicella: a ten-year case series and systematic review of the literature. Ital J Pediatr.

[43] Takanashi J, Miyamoto T, Ando N (2010). Clinical and radiological features of rotavirus cerebellitis. AJR Am J Roentgenol.

[44] Rossi A, Martinetti C, Morana G (2016). Neuroimaging of infection and inflammatory diseases of the pediatric cerebellum and brainstem. Neuroimag Clin N Am.

[45] Brisca G, La Valle A, Campanello C (2020). Listeria meningitis complicated by hydrocephalus in an immunocompetent child: case report and review of the literature. Ital J Pediatr.

