# Supplementary material for: Magnetic resonance imaging patterns of paediatric brain infections: a pictorial review based on the Western Australian experience
Source: Insights Imaging. 2022 Oct 4;13:160. doi: 10.1186/s13244-022-01298-1 (PMC9532482; doi:10.1186/s13244-022-01298-1)
Supplement: Supplementary file 1 — Additional file 1. Supplementary figures with ADC maps. [file 13244_2022_1298_MOESM1_ESM.pdf]

ELECTRONIC SUPPLEMENTARY MATERIAL

Magnetic Resonance Imaging Patterns of Paediatric Brain Infections: A Pictorial Review Based on the Western Australian Experience

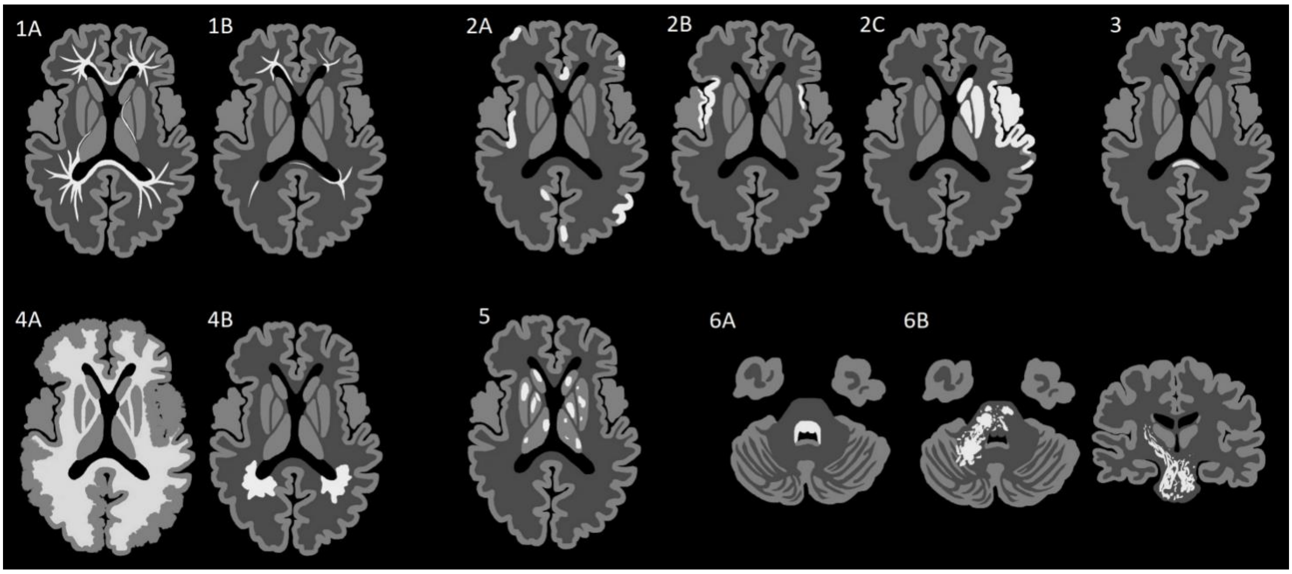

Figure 1: General MRI patterns of paediatric brain infections

Composite images of disease patterns overlaid on generic axial sections through the supratentorial brain (level of basal ganglia and 3rd ventricle) and infratentorial brain (mid-pons and 4th ventricle), as well as a coronal section through the basal ganglia and 3rd ventricle. Each key pattern on MRI has been allocated a number, with subdivisions indicated by a letter. Pattern 1: Restricted diffusion in supratentorial white matter (A - diffuse, B - limited). Pattern 2: Restricted diffusion in supratentorial grey matter injury (A - scattered, B - mesial temporal lobe(s), C - vascular territory). Pattern 3: Restricted diffusion in corpus callosum. Pattern 4: T2 hyperintensity in supratentorial white matter (A - with neuronal migration abnormality, B - without neuronal migration abnormality). Pattern 5: T2 hyperintensity in the basal ganglia and/or thalami. Pattern 6: T2 hyperintensity in the posterior fossa (A - dorsal pons, B - diffuse brainstem with longitudinal tract involvement).

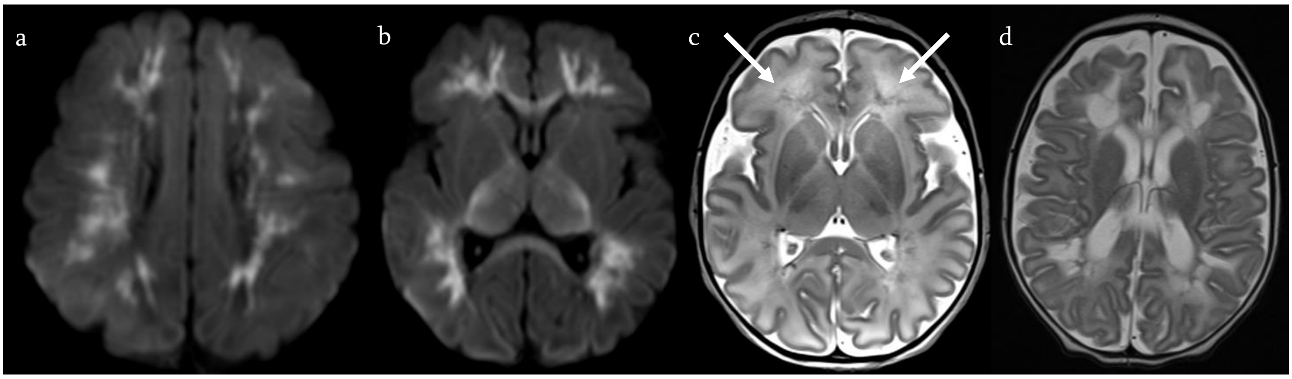

Figure 2: Restricted diffusion in supratentorial white matter - diffuse (Pattern 1A)

2-day-old term infant with generalised seizures and CSF-proven parechovirus encephalitis. DWI (a, b) demonstrated a striking pattern of restricted diffusion involving deep and periventricular white matter. On T2 weighted imaging (c), corresponding foci of low signal intensity were relatively subtle (arrows). A follow-up scan 5 weeks later (d) revealed significant tissue loss with areas of cystic encephalomalacia.

ADC maps for a) and b) in Figure 2.

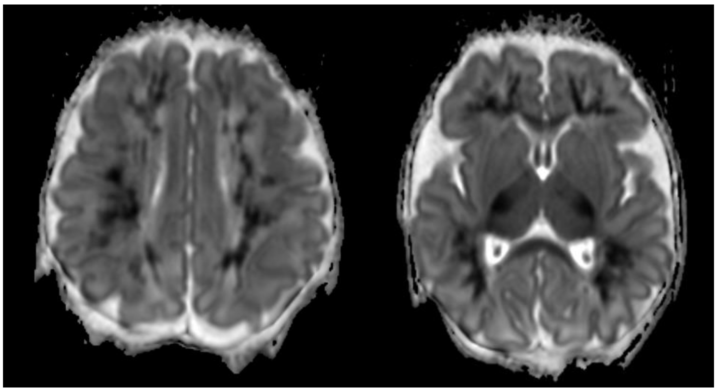

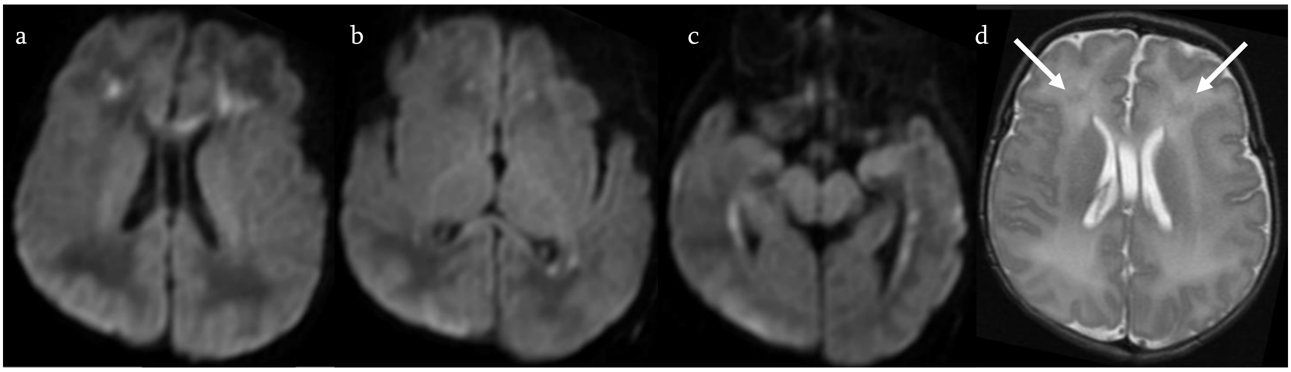

Figure 3: Restricted diffusion in supratentorial white matter - limited (Pattern 1B)

8-day-old term infant with raised inflammatory markers and vomiting (no seizures). CSF was positive for enterovirus. DWI (a, b, c) demonstrated scattered foci of restricted diffusion involving deep and periventricular white matter, best seen in the frontal lobes (a) and temporal lobes (c). On T2 weighted imaging (d), corresponding foci of low signal intensity were difficult to appreciate (arrows).

ADC maps for a), b) and c) in Figure 3.

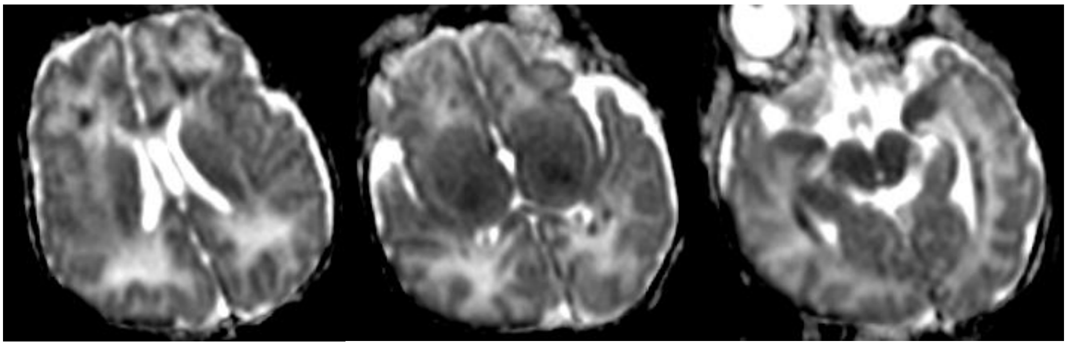

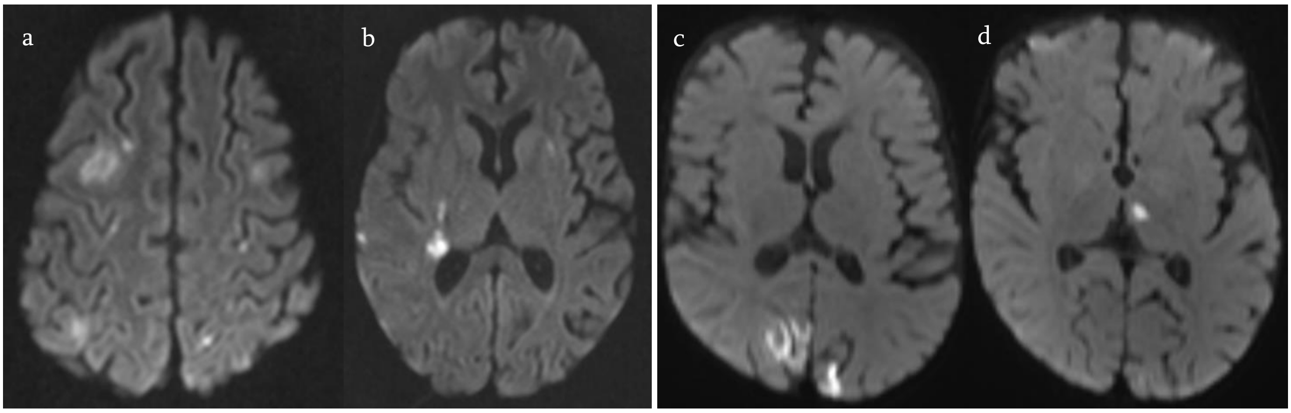

Figure 4: Restricted diffusion in supratentorial grey matter - scattered lesions (Pattern 2A)

Case 1: 4-year-old child with somnolence and hyperaesthesia, in the setting of acute lymphoblastic leukaemia (ALL) and *Scedosporium prolificans* fungaemia. DWI (a, b) demonstrated scattered foci of restricted diffusion, mainly affecting cortical grey matter with a bilateral though asymmetric distribution.

Case 2: 4-month-old infant with respiratory distress, in the setting of HIV, *Pneumocystis jiroveci* pneumonia and an abnormal cranial ultrasound. DWI (c, d) demonstrated scattered foci of restricted diffusion, mainly affecting cortical grey matter with a bilateral though asymmetric distribution.

ADC maps for Figure 4.

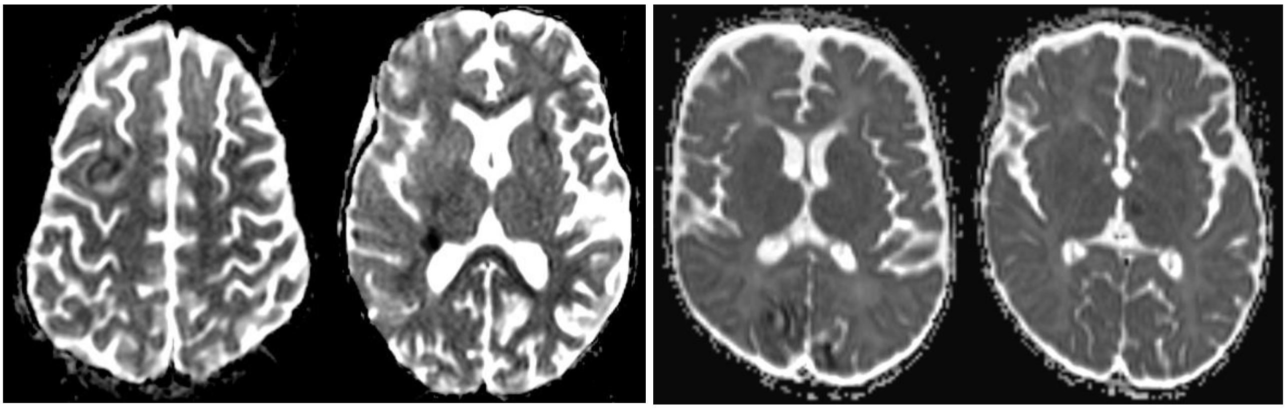

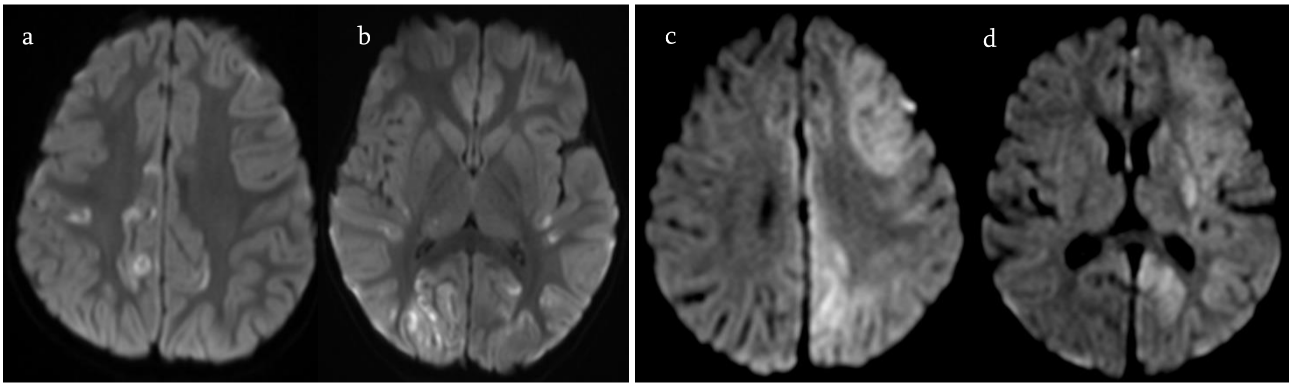

Figure 5: Restricted diffusion in supratentorial grey matter - scattered lesions (Pattern 2A)

Case 1: 3-year-old child with fever, headaches and reported visual loss. CSF was positive for HSV-1. DWI (a, b) demonstrated foci of restricted diffusion in cortical grey matter, with a bilateral though asymmetric distribution.

Case 2: 10-month-old infant with afebrile focal seizures. CSF was positive for HSV-1. DWI (c, d) demonstrated regions of confluent cortical restricted diffusion, with an asymmetric distribution.

ADC maps for Figure 5.

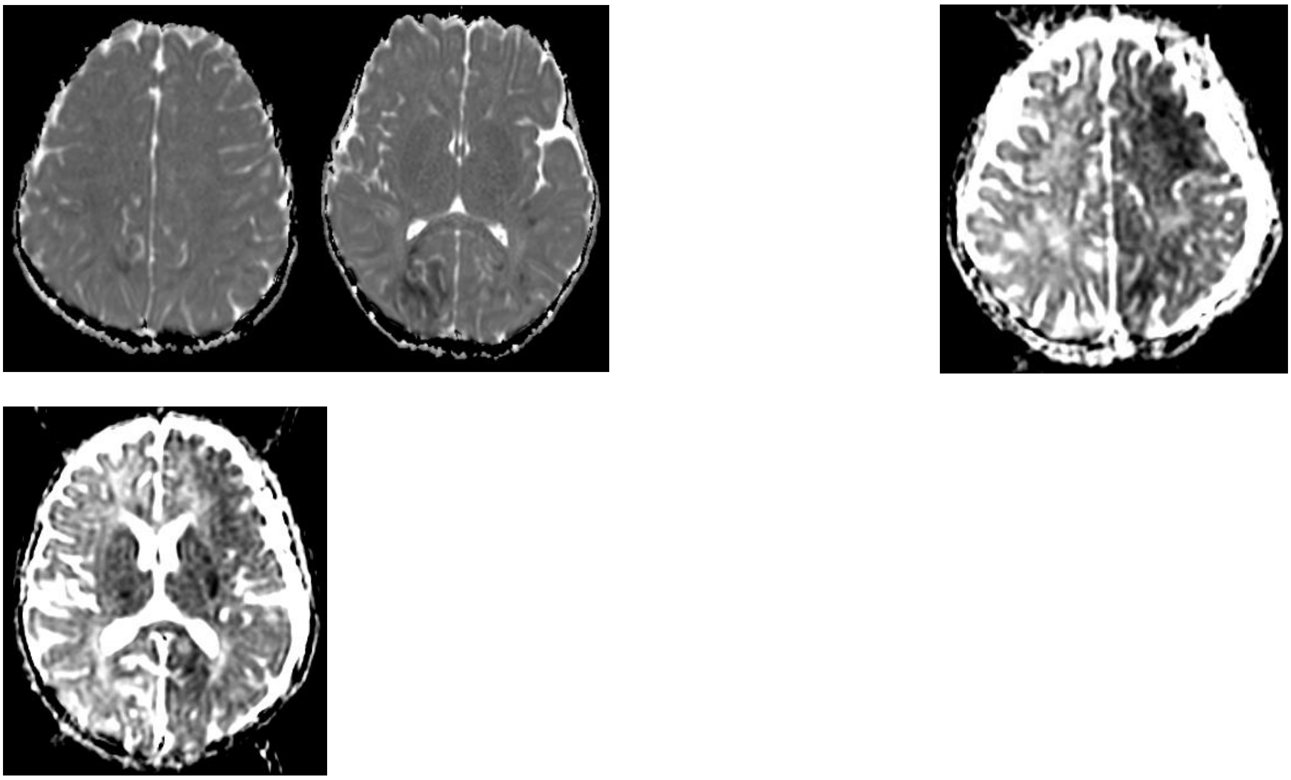

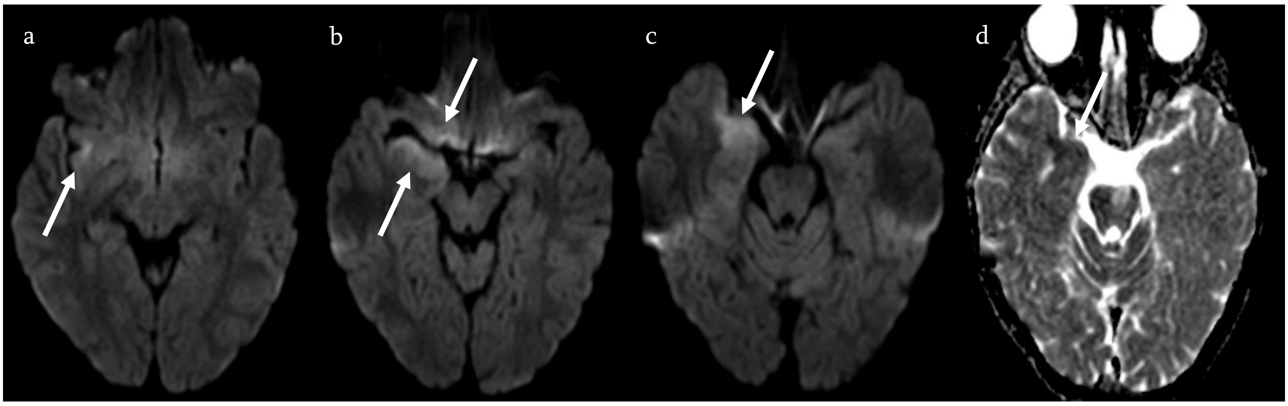

Figure 6: Restricted diffusion in supratentorial grey matter - mesial temporal lobe (Pattern 2B)

7-year-old child with headache, fluctuating consciousness and tonic-clonic seizures. CSF was positive for HSV-1. DWI (a, b, c) and ADC map (d) demonstrated asymmetric signal abnormalities predominantly involving the right mesial temporal lobe (amygdala and uncus), anterior perforated substance and insular cortex (arrows), as would be typical for HSV encephalitis in adults.

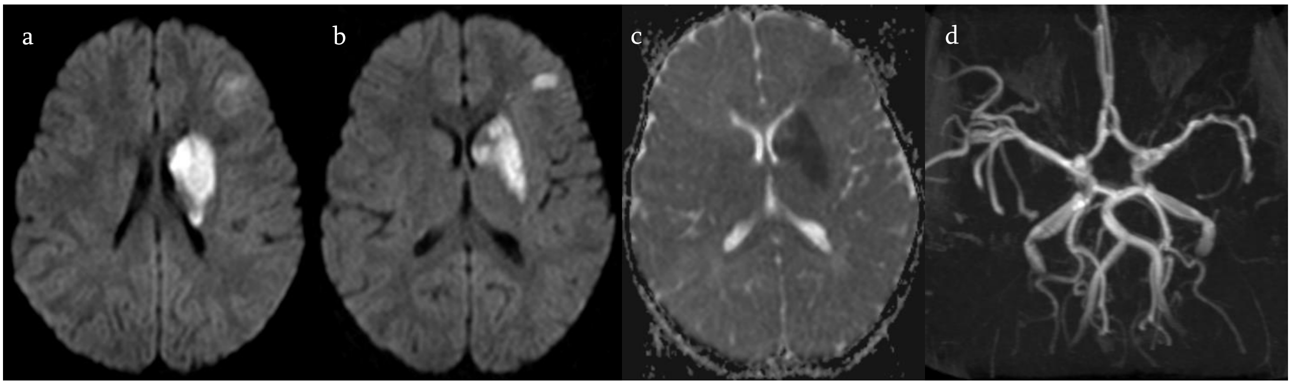

Figure 7: Restricted diffusion in supratentorial grey matter - vascular territory infarct (Pattern 2C)

2-year-old child with right upper and lower limb weakness in the setting of prior varicella infection (IgG positive), in keeping with post-varicella arteriopathy. DWI (a, b) and apparent diffusion coefficient (ADC) map (c) were consistent with an acute left caudato-lenticular infarct and a small infarct within the left frontal lobe. MR angiography (d) demonstrated left middle cerebral arteriopathy.

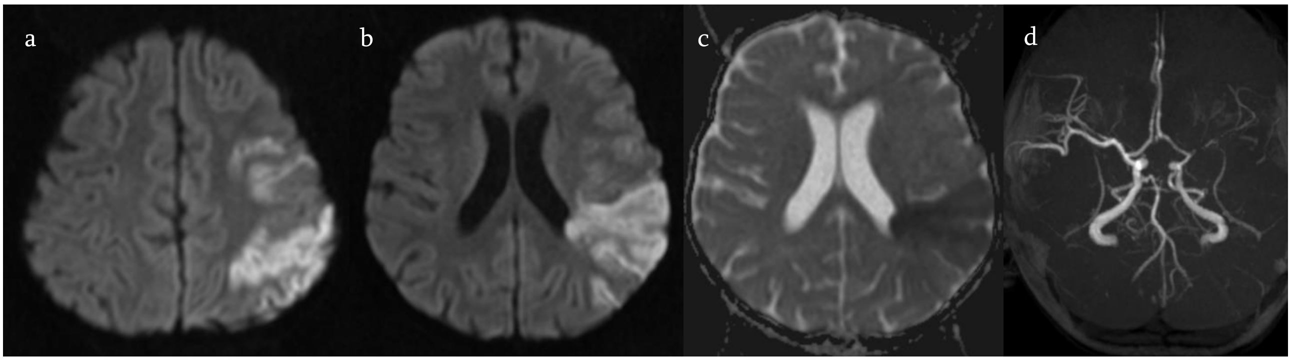

Figure 8: Restricted diffusion in supratentorial grey matter - vascular territory infarct (Pattern 2C)

4-year-old child with 2 weeks of malaise, followed by decreased conscious state and right arm weakness. CSF was positive for tuberculosis (TB). DWI (a, b) and ADC map (c) were consistent with an acute infarct involving the left frontoparietal lobes. MR angiography (d) demonstrated left middle cerebral arteriopathy.

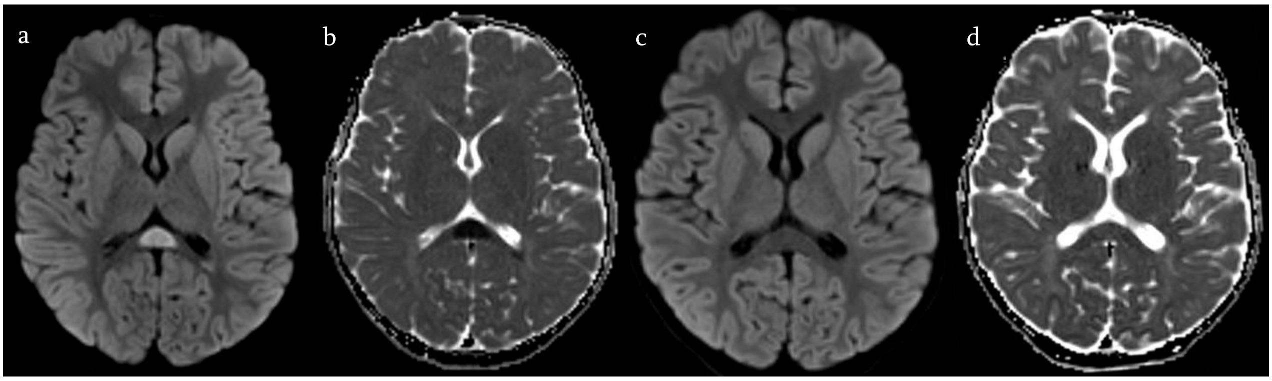

Figure 9: Restricted diffusion in corpus callosum (Pattern 3)

3-year-old child with gastrointestinal illness, followed by lethargy, irritability and suspected focal seizures. Stool was positive for norovirus. DWI (a) and ADC map (b) demonstrated a cytotoxic lesion midline within the splenium. Follow-up MRI 2 weeks later demonstrated complete resolution of the splenial lesion on DWI (c) and ADC (d) images.

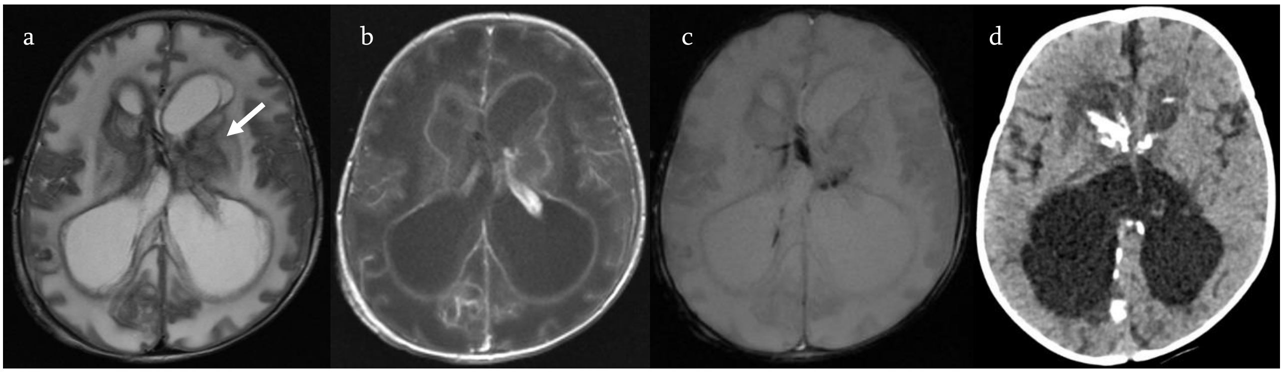

Figure 10: T2 hyperintensity in supratentorial white matter - with neuronal migration abnormality (Pattern 4A)

4-week-old infant with seizures, irritability and bulging fontanelles. CSF analysis was consistent with neurotoxoplasmosis. T2 weighted imaging (a) demonstrated diffuse white matter hyperintensity, widespread abnormal cortical development and hydrocephalus. Lesions were seen throughout the cerebral hemispheres, in the periventricular regions (arrow), basal ganglia and the thalami. Post-contrast T1 weighted imaging (b) demonstrated peripheral enhancement of parenchymal lesions, and ependymal enhancement in the lateral ventricles (reflecting ventriculitis). On a T2* weighted sequence (c), foci of susceptibility artefact corresponded to areas of haemorrhage and calcification (secondary to parenchymal necrosis). Non-contrast CT head performed 5 months later (d) better demonstrated regions of periventricular calcification.

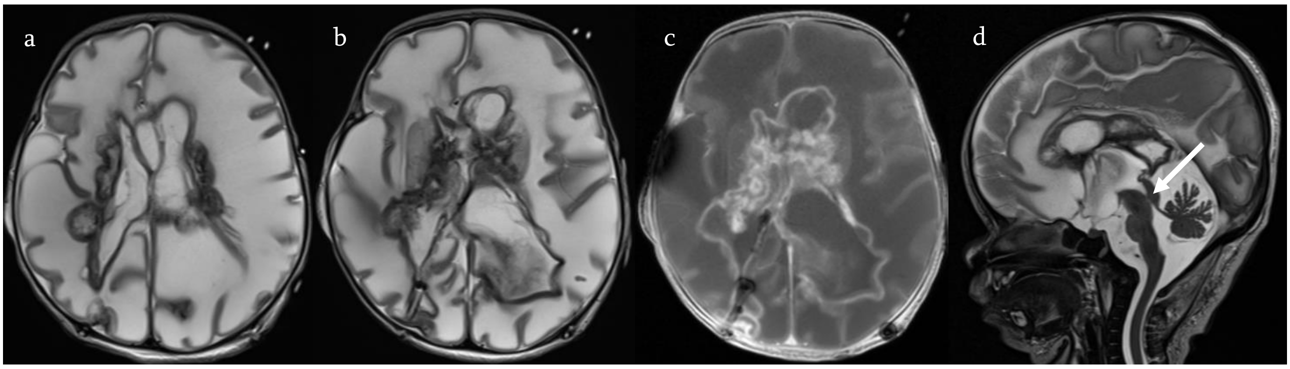

Figure 11: T2 hyperintensity in supratentorial white matter - with neuronal migration abnormality (Pattern 4A)

2-week-old infant with multiple seizures and a bulging anterior fontanelle. CSF analysis was consistent with neurotoxoplasmosis. T2 weighted images (a, b, d) demonstrated diffuse white matter injury, widespread abnormal cortical development and hydrocephalus relating to aqueduct stenosis (arrow). Post-contrast T1 weighted imaging (c) demonstrated multiple peripherally enhancing lesions in the basal ganglia and periventricular regions, as well as enhancement of the ependymal lining of the lateral ventricles (reflecting ventriculitis).

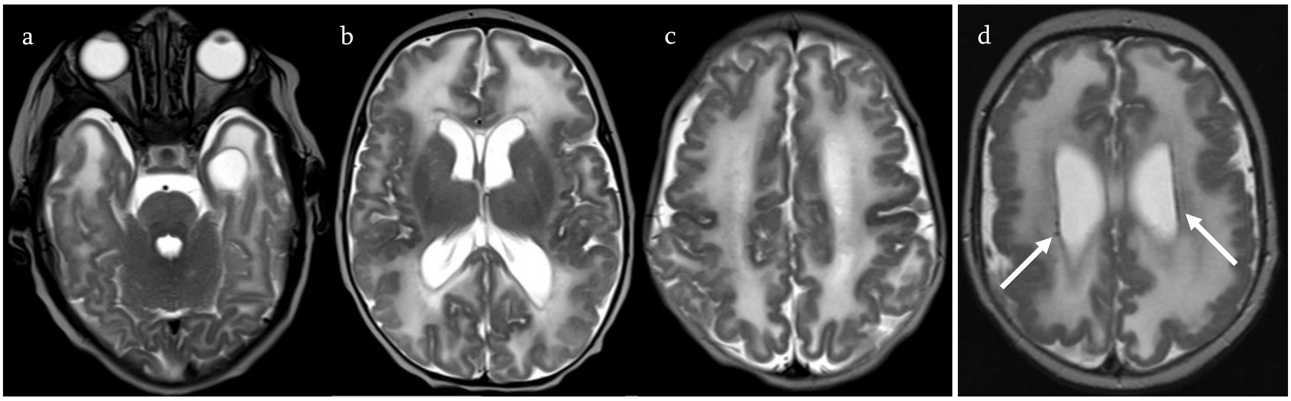

Figure 12: T2 hyperintensity in supratentorial white matter - with neuronal migration abnormality (Pattern 4A)

Case 1: 11-week-old infant with bilateral sensorineural hearing loss on newborn testing. Guthrie test was positive for CMV. T2 weighted imaging (a, b, c) demonstrated polymicrogyria with diffuse white matter hyperintensity and cystic change in the left anterior temporal pole. Clinical notes at 14 months of age indicated developmental delay.

Case 2: 4-month-old infant with microcephaly, developmental delay and hypertonia under investigation. Guthrie test was positive for CMV. T2 weighted imaging (d) demonstrated polymicrogyria with diffuse white matter hyperintensity and subtle periventricular calcifications (arrows).

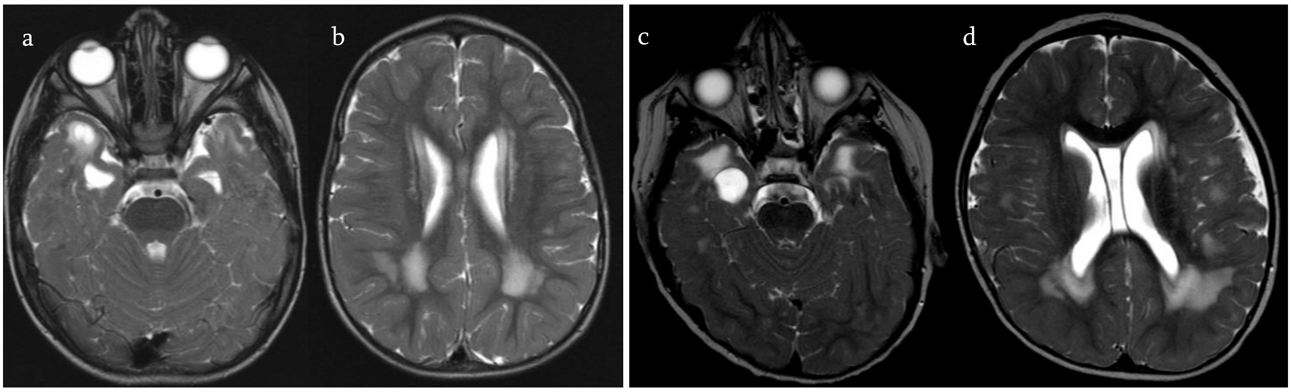

Figure 13: T2 hyperintensity in supratentorial white matter - without neuronal migration abnormality (Pattern 4B)

Case 1: 2-year-old child with congenital left sensorineural hearing loss. Guthrie test was positive for CMV. T2 weighted imaging (a, b) demonstrated cystic change at the right anterior temporal pole, with bilateral parietal and periventricular white matter hyperintensity.

Case 2: 2-year-old child with developmental delay. Guthrie test was positive for CMV. T2 weighted imaging (c, d) demonstrated cystic change at the right anterior temporal pole, with bilateral peritrigonal white matter hyperintensity.

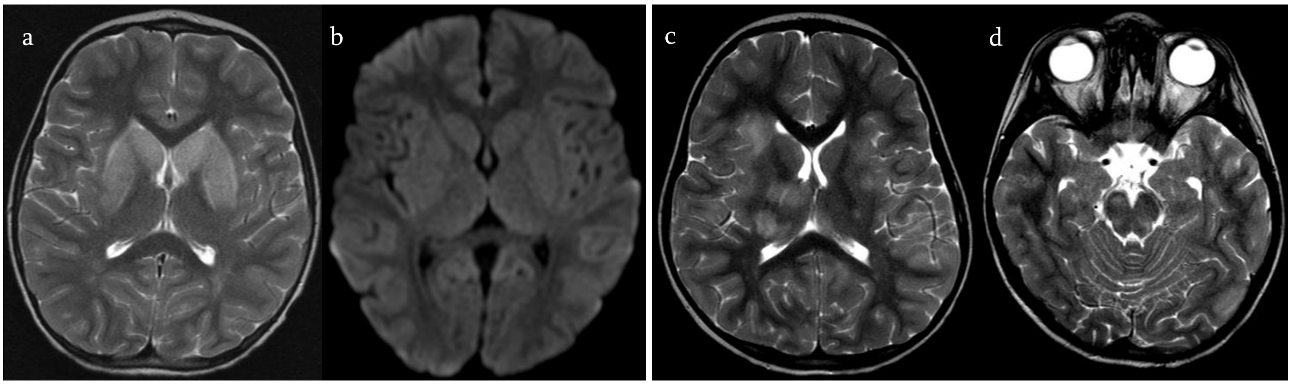

Figure 14: T2 hyperintensity in basal ganglia and/or thalami (Pattern 5)

Case 1: 5-year-old child with afebrile generalized tonic-clonic seizures and CSF proven EBV encephalitis. T2 weighted imaging (a) demonstrated bilateral hyperintensity of the basal ganglia, without abnormality on DWI (b).

Case 2: 4-year-old child with fever, sore throat, irritability and CSF proven EBV encephalitis. T2 weighted imaging (c, d) demonstrated bilateral hyperintensity of the basal ganglia and thalami (c), as well as scattered lesions in the cerebrum and brainstem (d). No DWI abnormality was present (not shown).

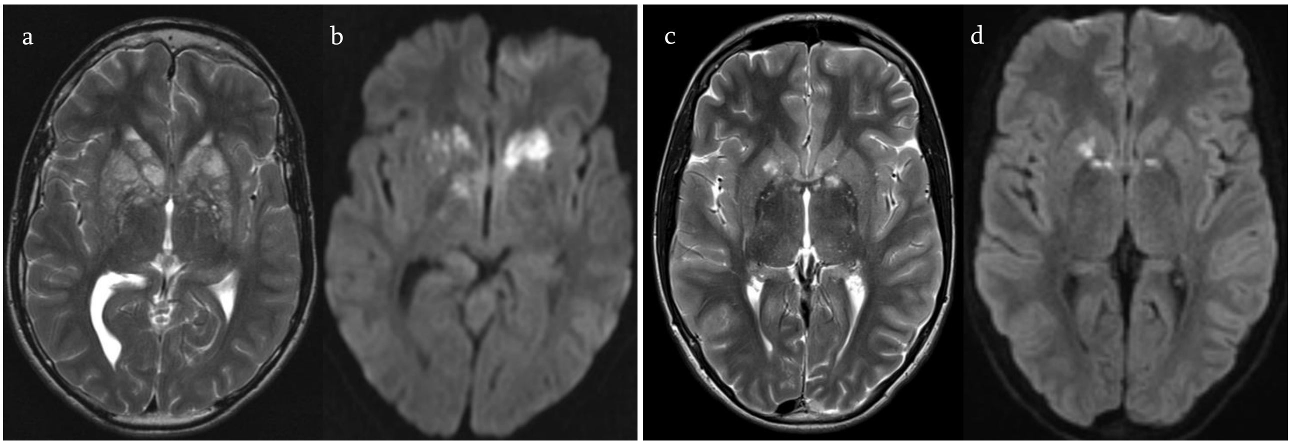

Figure 15: T2 hyperintensity in basal ganglia and/or thalami (Pattern 5)

Case 1: 15-year-old child with headache, nausea and vomiting. CSF was positive for cryptococcus. T2 weighted imaging (a) demonstrated bubble-like lesions within the basal ganglia (relating to gelatinous pseudocysts), with a relatively smaller component of restricted diffusion (b).

Case 2: 14-year-old child with malaise for 2 weeks. CSF was positive for cryptococcus. T2 weighted imaging (c) demonstrated bubble-like lesions within the basal ganglia and thalami, with a relatively smaller component of restricted diffusion (d).

ADC maps for b) and d) in Figure 15.

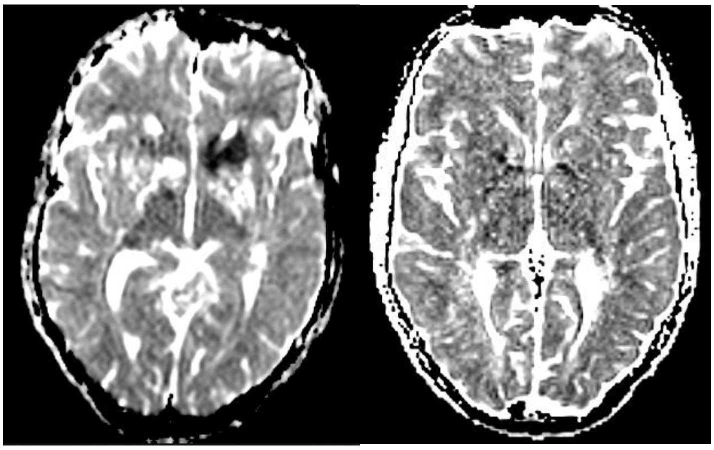

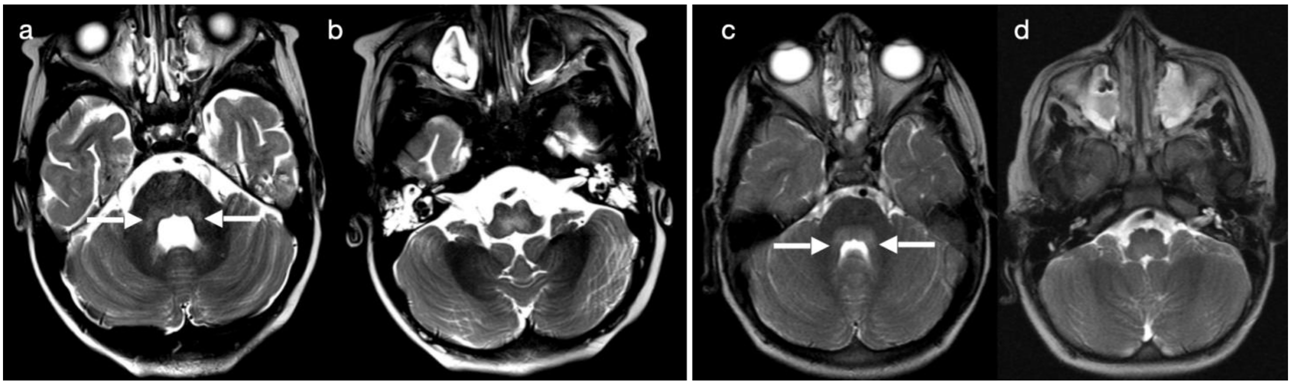

Figure 16: T2 hyperintensity in posterior fossa - dorsal pons (Pattern 6A)

Case 1: 4-year-old child with fluctuating GCS. Stool was positive for enterovirus (serotype E-71). T2 weighted imaging (a, b) demonstrated hyperintensity of the dorsal pons and medulla, with extension of signal abnormality into the middle cerebellar peduncles bilaterally (arrows).

Case 2: 3-year-old child with lethargy, vomiting and fever, as well as right facial nerve palsy and unsteadiness on feet. Stool was positive for enterovirus (serotype not known). T2 weighted imaging (c, d) demonstrated hyperintensity of the dorsal pons and medulla, with extension of signal abnormality into the middle cerebellar peduncles bilaterally (arrows).

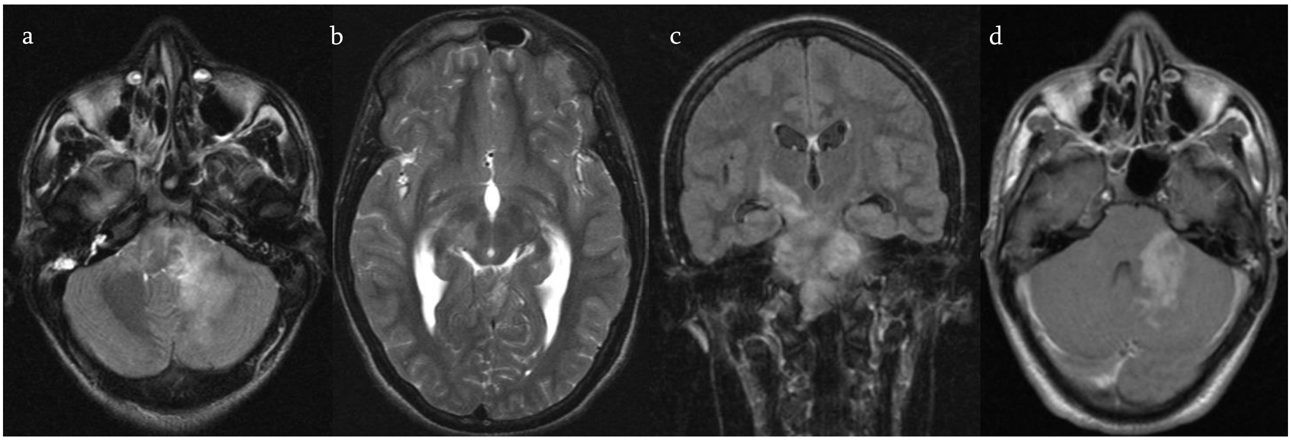

Figure 17: T2 hyperintensity in posterior fossa - diffuse brainstem with longitudinal tract involvement (Pattern 6B)

14-year-old child with headache, vomiting, left cerebellar signs, left upper motor neuron facial palsy, diplopia and slurred speech. T2 weighted images (a, b) demonstrated asymmetric T2 hyperintensity within the brainstem and left cerebellar hemisphere. Coronal FLAIR image (c) demonstrated extension of abnormality along white matter tracts to the contralateral supratentorial brain. Patchy enhancement was appreciable on post-contrast T1 weighted imaging (d). Minimal DWI change was seen (not shown). Neuromelioidosis was proved on brain tissue sampling.

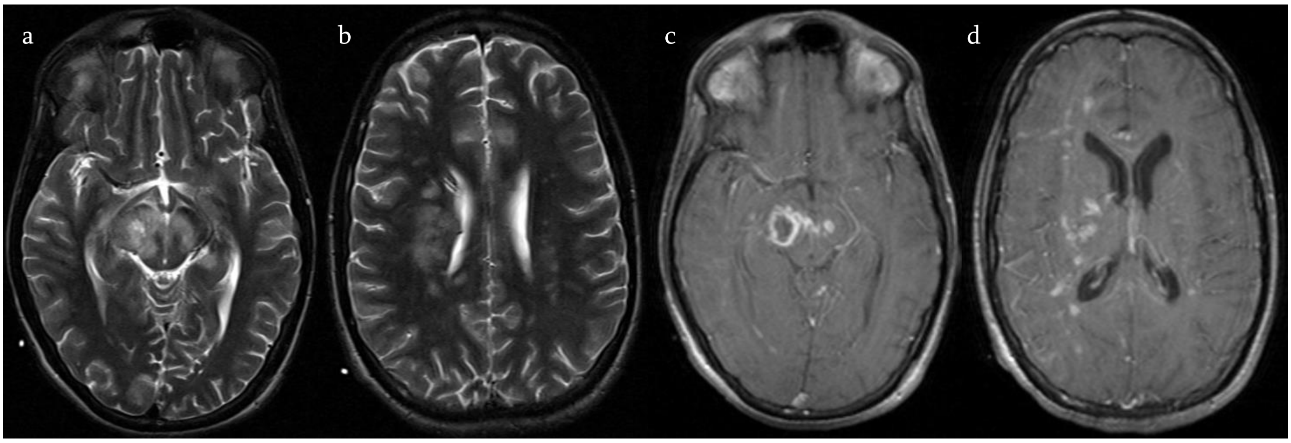

Figure 18: T2 hyperintensity in posterior fossa - diffuse brainstem with longitudinal tract involvement (Pattern 6B)

Follow-up MRI after 2 weeks (same patient as Figure 17 with biopsy-proven neuromelioidosis) showed T2 hyperintensities (a, b) following longitudinal white matter tracts into the supratentorial brain. Post-contrast T1 weighted imaging (c, d) demonstrated a rim-enhancing abscess in the right cerebral peduncle and multiple enhancing foci in the supratentorial brain (representing micro-abscesses).

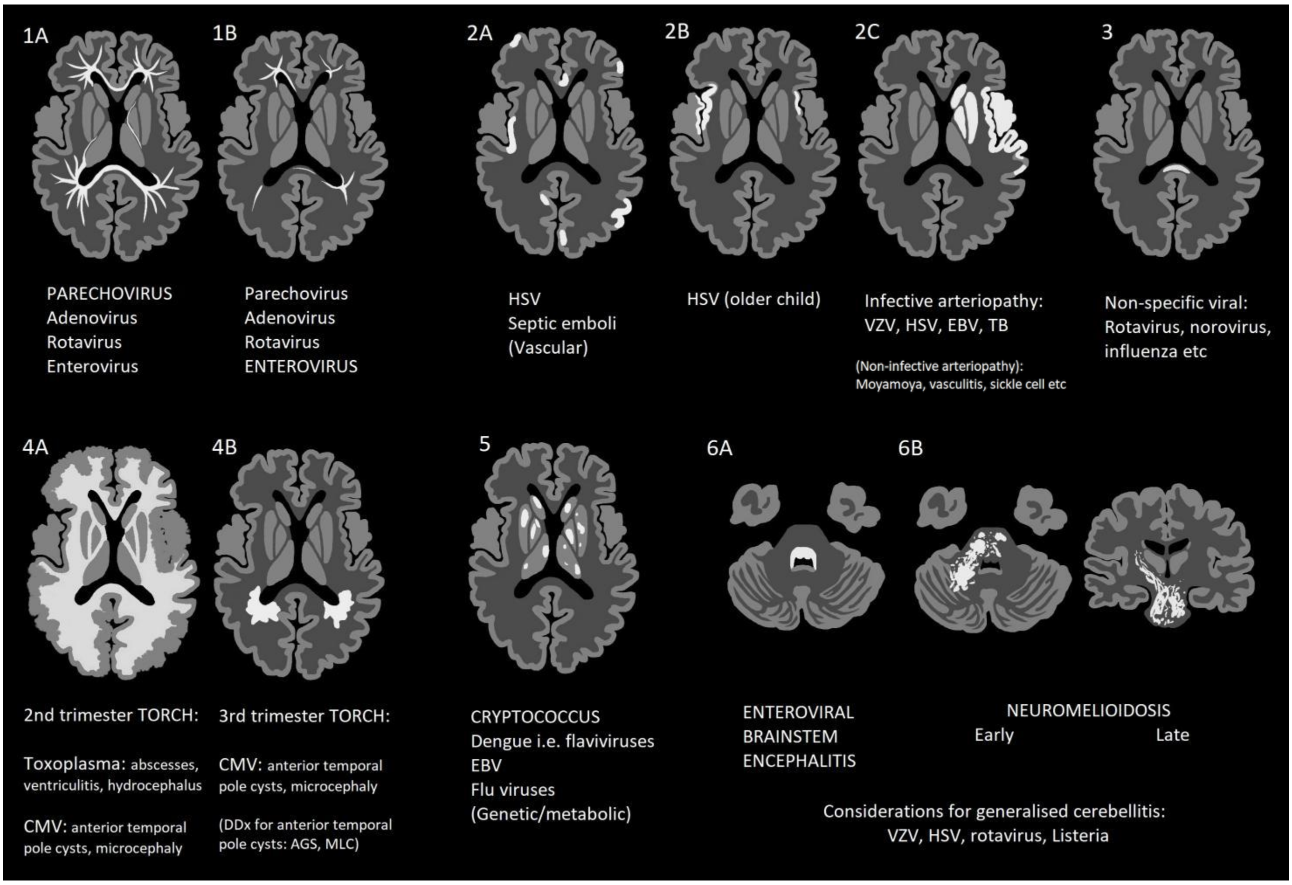

Figure 19: MRI patterns of paediatric brain infections, with differentials relevant to the WA environment

A summary of the key MRI patterns and their sub-patterns as discussed in this review, with the causative pathogens tailored to the WA environment.
